# Clinical characteristics, risk factors and outcome of severe Norovirus infection in kidney transplant patients: a case-control study

**DOI:** 10.1186/s12879-021-06062-2

**Published:** 2021-04-15

**Authors:** Julien Gras, Moustafa Abdel-Nabey, Axelle Dupont, Jérôme Le Goff, Jean-Michel Molina, Marie Noëlle Peraldi

**Affiliations:** 1 Infectious Diseases Department, APHP, Saint-Louis Hospital, Paris, France; 2 INSERM U944, “Cellular Biology of Viral Interactions” Team, Université de Paris, Paris, France; 3 Nephrology and Kidney Transplant Department, APHP Saint Louis Hospital, Paris, France; 4 Biostatistics and Medical IT Department, APHP- Saint-Louis Hospital, Paris ECSTRA Team, UMR 1153 INSERM, Université de Paris, Paris, France; 5 Virology Department, APHP, Saint-Louis Hospital, Paris, France

**Keywords:** Norovirus, Diarrhea, Kidney transplantation, Graft function, Risk factors

## Abstract

**Background:**

Human Norovirus (HuNoV) has recently been identified as a major cause of diarrhea among kidney transplant recipients (KTR). Data regarding risk factors associated with the occurrence of HuNoV infection, and its long-term impact on kidney function are lacking.

**Methods:**

We conducted a retrospective case-control study including all KTR with a diagnosis of HuNoV diarrhea. Each case was matched to a single control according to age and date of transplantation, randomly selected among our KTR cohort and who did not develop HuNoV infection. Risk factors associated with HuNoV infection were identified using conditional logistic regression, and survival was estimated using Kaplan-Meier estimator.

**Results:**

From January 2012 to April 2018, 72 cases of NoV diarrhea were identified among 985 new KT, leading to a prevalence of HuNoV infection of 7.3%. Median time between kidney transplantation and diagnosis was 46.5 months (Inter Quartile Range [IQR]:17.8–81.5), and the median duration of symptoms 40 days (IQR: 15–66.2). Following diagnosis, 93% of the cases had a reduction of immunosuppression. During follow-up, de novo Donor Specific Antibody (DSA) were observed in 8 (9%) cases but none of the controls *(p* = 0.01). Acute rejection episodes were significantly more frequent among cases (13.8% versus 4.2% in controls; *p* = 0,03), but there was no difference in serum creatinine level at last follow-up between the two groups (*p* = 0.08). Pre-transplant diabetes and lymphopenia below 1000/mm^3^ were identified as risks factors for HuNoV infection in multivariate analysis.

**Conclusion:**

HuNoV infection is a late-onset and prolonged infection among KTR. The current management, based on the reduction of immunosuppressive treatment, is responsible for the appearance of de novo DSA and an increase in acute rejection episodes.

## Introduction

Kidney transplantation (KT) is now considered as the standard of care for the treatment of chronic kidney disease. The success rate of KT has been greatly improved thanks to new immunosuppressive protocols including calcineurin inhibitors (CNIs), with a graft survival up to 90% after 5 years [1–3]. However, immunosuppressive therapies increase the risk of infections, which now represent one of the leading causes of death among kidney transplant recipients (KTR), especially in older patients with underlying comorbidities [4]. In particular, CNIs inhibit LT cell response which plays a key role in antiviral immunity [5]. KTR are therefore highly susceptible to the reactivation of latent virus including CMV or BK virus, as well as de novo viral infections such as Human Norovirus (HuNoV) infection.

Chronic diarrhea is a frequent complication following KT, with a cumulative incidence of 23% at 3 years, and is associated with significant morbidity. Although some medication (including immunosuppressants) can elicit diarrhea, infections are responsible for almost half of the cases of diarrhea [6–8]. Thanks to the improvement of microbiological diagnostic methods, a large proportion of undocumented or presumably drug-induced diarrhea have been attributed to infectious agents, especially norovirus [7, 8].

Human noroviruses (HuNoV) are genetically diverse non-enveloped viruses part of the *Caliciviridae* family, with a single-strand RNA genome of approximately 7.5 kb in length. Based on the aminoacid diversity of the complete VP1 gene, HuNoV have recently been segregated in 10 different genogroups (and further 49 genotypes), genogroups I and II containing the majority of strains associated with human disease [9]. Noroviruses represent one of the leading cause of acute gastroenteritis worldwide across all age groups, with an estimated 70.000 to 200.000 deaths annually [10]. Among immunocompetent individuals, HuNoV are responsible for mild gastroenteritis, usually resolving spontaneously in two to 3 days [11]. However, in immunosuppressed individuals and especially solid organ transplant recipients (SOTR), HuNoV infection may present as a severe acute diarrhea and progress to chronic infection with or without clinical symptoms, due to persistant virus shedding [12, 13].

Among KTR, cohort studies have shown that HuNoV represents the second most frequent pathogen identified in case of diarrhea, with a proportion varying between 16.7 and 35% [8, 14, 15]. In this population, HuNoV infection is a late-onset complication and is frequently associated with severe weight loss and the fluctuation of CNI serum levels [8, 15]. Moreover, HuNoV constitutes one of the main etiology of chronic diarrhea with an average duration of symptoms of 8.7 months [8, 13]. There is no specific antiviral treatment, and current management of HuNoV diarrhea relies only on the reduction of immunosuppressive therapy especially mycophenolate mofetil (MMF) and CNI, despite the risk of acute rejection and log-term graft failure [16]. To date, despite extensive descriptive data regarding clinical presentation and therapeutic management of HuNoV diarrhea among SOTR, available studies have found conflicting results concerning the impact of HuNoV infections on graft function, mostly depending on the duration of follow-up [15, 17]. Moreover, only two case-control studies have focused on potential risk factors associated with the occurrence of HuNoV infection among KTR. In the first one, Brakemeier et al. identified immunosuppression containing steroids and antirejection therapy as risk factors for HuNoV diarrhea [18], wheras Rolak et al. did not find any in their multivariate analysis [17].

We therefore conducted a restropective case-control study to describe the clinical characteristics of HuNoV diarrhea among a large cohort of KTR, evaluate its impact on long-term graft survival, and identify potential risk factors associated with HuNoV infection in this context.

## Methods

### Study population

In this retrospective study, we included all KTR followed in the Nephrology and Kidney Transplantation unit at APHP-Saint Louis hospital (Paris, France), who were diagnosed with HuNoV diarrhea from January 2012 to April 2018. All patients provided written informed consent at the time of transplant to be included in the database.

Cases of HuNoV diarrhea were identified by screening the local transplant and microbiology databases, and defined by a positive stool sample after specific RT-qPCR testing (see below). For each case, one control was randomly selected among the KTR cohort from the same hospital, and matched (1:1 ratio) according to the following criteria: age at the time of KT (+/− 1 year), and date of transplantation (+/− 1 year). The only exclusion criteria for the control group was the diagnosis of HuNoV infection. Controls were not excluded if they presented other types of diarrhea or infectious complications.

### Evaluation for HuNoV infection

The diagnosis of diarrhea was defined by the occurrence of three or more watery stools per day. In case of diarrhea, all KTR were routinely tested for the following pathogens: 1) bacterial pathogens in stool cultures using standard media (*Campylobacter* sp., *Yersinia* sp., *Shigella* sp., *Salmonella* sp.); 2) *Clostridium difficile* by toxin-B gene RT-PCR assay; 3) CMV whole blood replication by PCR (ABBOT CMV real time kit); 4) viruses by testing stool for the detection of Adenovirus, Rotavirus and Norovirus-1 and 2 by RT-PCR (RIDA®GENE viral Stool Panel, R-Biopharm, Germany). The NoV RT-PCR allowed the detection of genogroups I and II using primers targeting the ORF1/ORF2 region, with a lower limit of detection of 50 copies/reaction. The recurrence of HuNoV diarrhea was defined by the regression of clinical symptoms followed by a new stool sample positive for HuNoV testing.

### Data collection and definitions

For both cases and controls, the following data were retrospectively collected using a standard case report form: 1) demographic features (age, sex, underlying comorbidities such as diabetes); 2) pre-transplant data: primary kidney disease, duration of dialysis (months), calculated Panel Reactive Antibody (cPRA); 3) characteristics of KT: type of donor, immunosuppressive regimen (induction and maintenance therapies), HLA mismatch. The occurrence of whole-blood CMV replication and/or other opportunistic infections (OI) and acute rejection episodes between KT and HuNoV infection were also recorded.

For each case, the following data on HuNoV infection were described: 1) median time between KT and diagnosis; 2) immunosuppressive regimen at the time of diagnosis (number and name of immunosuppressive drugs, dosage and serum levels if available); 3) clinical parameters: percentage of weight loss, duration of diarrhea; 4) laboratory data at the time of diagnosis: serum creatinine level (μmol/L), lymphocyte count (/mm^3^) and gamma-globulin level (g/L) if available as serum immunoglobulin levels were not routinely obtained in our institution; 5) occurrence of acute kidney injury following HuNoV diarrhea according to the 2012 KDIGO classification [19].

### Follow-up and outcome

For follow-up and outcome analysis, we defined for each control an index date corresponding to the time point with the same delay after KT as HuNoV diagnosis of the matched case. The following events occurring after HuNoV infection (or the index date for controls) were recorded until last encounter recorded in the patient’s medical record: 1) modification of the immunosuppressive regimen; 2) long-term impact on graft function assessed by the following criteria: serum creatinine levels (μmol/L), appearance of donor-specific antibodies (DSA) which can promote antibody-mediated rejection of solid organ allografts [20], occurrence of biopsy proven acute rejection according to the BANFF criteria [16]; 3) return to dialysis; 4) death.

### Statistical analysis

Continuous variables are presented as medians and interquartile ranges (IQR) and categorical variables as numbers and percentages. Survival and event-free survival curves were obtained using Kaplan-Meier plots with censoring for loss to follow-up or end of observation. Comparison between cases and controls were made using log-rank tests. Risk factors associated with the occurrence of HuNoV diarrhea were identified using univariate exact conditional logistic regression, by including only the couples without any missing data in the analysis. Identified risk factors in univariate analysis are presented in terms of odd ratios (OR) and their 95% confidence interval (95% CI). Only the risk factors with a *p*-value below 0.1 were included in a multivariate model. All statistical tests were two-sided; *p*-values of < 0.05 were considered to be significant. Statistical analyses were performed using R software version 3.2.0 (http://www.R-project.org).

## Results

### Description of the population

From January 2012 to April 2018, 72 KTR with at least one episode of HuNoV diarrhea were identified. During the same period, 985 new KT were performed in our center, leading to a frequency of NoV infection of 7.3%. Seventy-two controls were matched in a 1:1 ratio by age and date of transplant. Demographic and transplant characteristics of cases and matched controls are described in Table 1.

**Table 1:** Characteristics of the NoV cases and matched controls

Variable	Norovirus cases (*n* = 72) *n* (%); median [IQR]	Controls (*n* = 72) *n* (%); median [IQR]	*p*-value
***Demographic features***			
Age at the time of KT *(years)*	46 [33.7–58.7]	46 [34–58.1]	0.83
Male sex	39 (54.2)	37 (51.4)	0.74
Primary kidney disease			0.08
Diabetic	15 (20.8)	7 (9.7)	
Vascular	11 (15.3)	13 (18.1)	
Chronic glomerulonephritis	11 (15.3)	19 (26.4)	
Others	35 (48.6)	33 (45.8)	
Diabetes			
Before transplantation	20 (27.8)	7 (9.7)	0.01
NODAT	4 (5.8)	5 (6.9)	1
***Characteristics of KT***			
c-PRA	30.1	30.2	0.88
First Transplantation	65 (90.3)	61 (84.7)	0.8
Deceased donor	61 (84.7)	60 (83.3)	1
Duration of dialysis *(months)*	28 [7–66]	24 [9–54]	0.24
HLA mismatch			0.97
< 2	11 (15.7)	10 (14.1)	
≥ 3	61 (84.3)	62 (85.9)	
Induction therapy			
ATG	70 (97)	67 (93)	0.55
Basiliximab	2 (3)	5 (7)	0.21
Maintenance therapy			
Dual therapy	29 (40.3)	30 (41.7)	1
Triple therapy	89.5 [68–118]	92 [72–123]	0.42
Events between KT and NoV diagnosis (or index date)			
Plasma CMV reactivation	14 (20)	10 (14.1)	0.38
Other OI ^a^	5 (7)	1 (1.4)	0.12
***Data recorded at diagnosis***			
Immunosuppressive regimen			
Corticosteroid	43 (59.7)	42 (58.3)	1
Mycophenolic acid	68 (94.4)	67 (93.1)	1
Tacrolimus	43 (59.7)	39 (54.2)	0.61
Ciclosporin	21 (29.2)	31 (43.1)	0.05
Laboratory data at diagnosis			
Serum creatinine level *(μM)*	139 [105–169]	135 [91–172]	0.95
eGFR *(ml/min/1.73m*^*2*^*)*	47 [41–65]	47 [38–68]	0.79
Positive CMV PCR	10 (13.8)	6 (8.3)	0,06
Gamma-globulin < 6.5 g/L	10 (13.9)	4 (5.6)	0.06
Lymphocyte count *(/mm*^*3*^*)*	500 [300–840]	875 [665–1430]	0.01

*Abbreviations*: *ATG* Anti-Thymocyte Globulin, *eGFR* estimated Glomerular Filtration Rate (MDRD), *KT* Kidney transplantation, *NODAT* New Onset Diabetes After Transplantation, *OI* Opportunistic infection, *PCR* Polymerase Chain Reaction, *cPRA* calculated Panel Reactive Antibody

^a^ Other OI: PTLD EBV-positive (Post-Transplantation Lymphoproliferative Disorder) (*n* = 2), tuberculosis (*n* = 1), nocardiosis (*n* = 1), invasive aspergillosis (*n* = 1), pneumocystis pneumonia (*n* = 1)

Demographic features did not differ between the two groups, except for pre-transplant diabetes which was statistically more frequent among cases (*N* = 20, 27.8%) compared to controls (*N* = 7, 9.7%; *p* = 0.01). Characteristics of KT were similar between cases and controls. In accordance with local institutional protocols, induction therapy was based on anti-thymocyte globulins in respectively 97 and 93% of the patients, except for high-immunological risk patients who received basiliximab. Maintenance therapy consisted in a dual therapy associating a CNI (tacrolimus or ciclosporin A) and mycophenolate mofetil (MMF) in 40.3% of the cases and 41.7% of the controls, and on a triple therapy with addition of corticosteroids for the other patients. Following KT, plasma CMV replication and other opportunistic infections occurred more frequently observed among cases, although the difference was not significantly different compared to controls.

### Characteristics of the patients with NoV diarrhea

Median time between KT and HuNoV infection was 46.5 months (IQR, 17.8–81.5). At the time of diagnosis, immunosuppressive regimen among HuNoV cases did not differ from matched controls at the index date (Table 1).

Median duration of the symptoms in cases was 40 days (IQR, 15–66), and diarrhea was associated weight loss in 46/72 (63.9%) of the patients. At the time of diagnosis, laboratory data showed an impaired graft function in HuNoV cases with a median serum creatinine level of 139 μM (IQR, 105–169), but not significantly different compared to controls (135 μM, IQR:91–172; *p* = 0.95). Median lymphocyte count was significantly lower in cases (500/mm^3^, [IQR: 300–840]) compared to controls (875/mm^3^, [IQR: 665–1430], *p* = 0.01). Gamma-globulin levels below 6.5 g/L were recorded in 10 (13.7%) cases versus 4 (5.6%) controls (*p* = 0.06, non significant) (Table 1).

Following HuNoV infection, 61% of patients with HuNoV infection developed acute kidney injury in the first month (due to dehydration and/or CNI overload), including one patient who needed renal replacement therapy. Immunosuppressive regimen was modified in 67 of the 72 cases. More specifically, MMF dose was reduced in 22 (30.5%) patients, withdrawn in 23 (32%), or replaced by azathioprine for 20 (28%) of them. In two patients, tacrolimus was stopped. Seven patients received intravenous polyvalent immunoglobulins specifically for the treatment of HuNoV diarrhea.

### Outcome and follow-up

Median duration of follow-up after HuNoV infection was 23.8 (IQR, 9.2–40.6) months for cases and 32 (IQR, 16.6–50) months for controls (*p* = 0,02) (Table 2).

**Table 2:** Outcome and follow-up

Variable	Norovirus cases (*n* = 72) *n* (%); median [IQR]	Controls (*n* = 72) *n* (%); median [IQR]	*p*-value
Median duration of follow-up *(months)*	23.8 [9.2–40.6]	32 [16.6–50]	0.02
Kidney function at last follow-up			
Serum creatinine *(μM)*	182 [121–231]	146 [114–202]	0.08
eGFR *(ml/min/1.73m*^*2*^*)*	38 [24–53]	44 [30–61]	0.16
Immunological outcome			
Number of patients with significant de novo DSA *(MFI > 3000)*	8 (11)	0	0.01
Acute rejection	10. (13.8)	3 (4.2)	0.03
Antibody-mediated rejection	7 (9.7)	2 (2.8)	
Mixed rejection	3 (4.2)	1 (1.4)	
Return to dialysis	8 (10.7)	5 (6.9)	0.07
Death	3 (4.2)	1 (1.4)	0.61

*Abbreviations*: *eGFR* estimated Glomerular Filtration Rate (MDRD), *MFI* Mean Fluorescence Intensity

Among cases, 18 (25%) patients had a recurrence of HuNoV diarrhea, in a median time of 9.4 (IQR, 7.7–23.4) months after the first episode. None of the seven patients who were initially treated with polyvalent immunoglobulins had any recurrence.

**Fig. 1:**
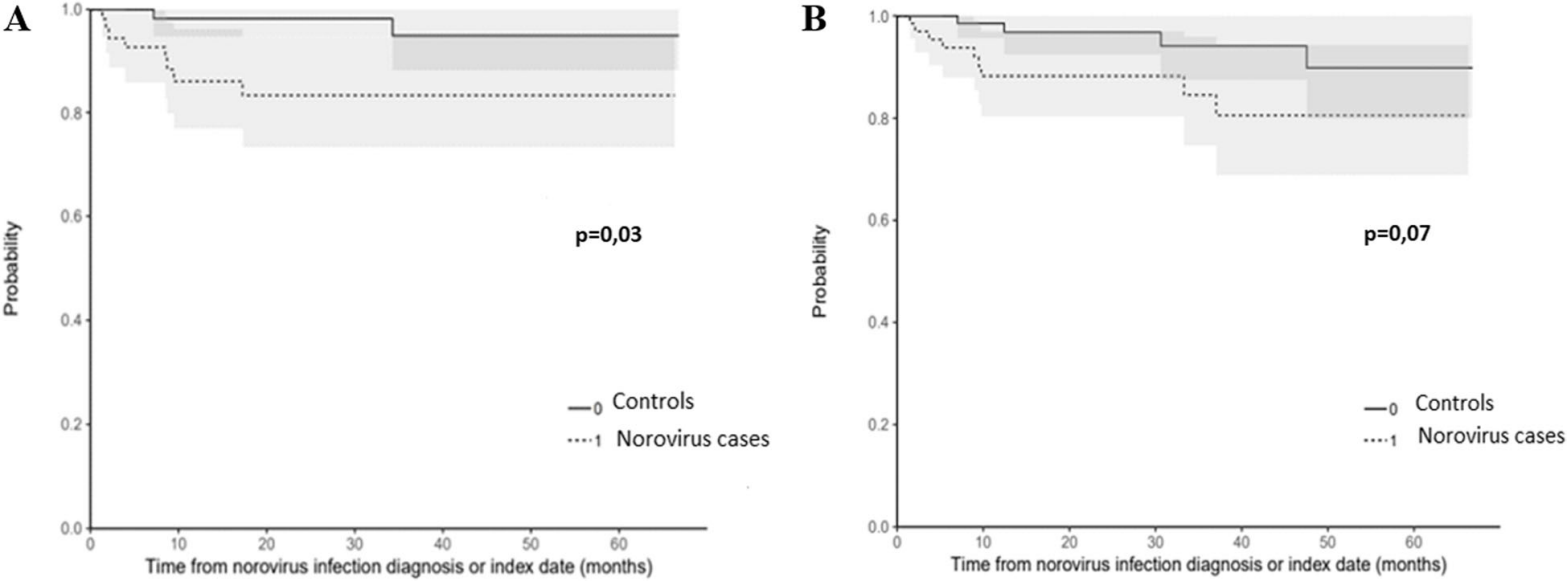
Comparative event-free survival curves between cases and controls for acute rejection episodes (**a**) and return to dialysis (**b**). Survival and event-free survival curves were obtained using Kaplan-Meier plots with censoring for loss to follow-up or end of observation for survival without rejection (**a**) and survival without dialysis (**b**). Comparison between cases and controls were made using log-rank tests

At the time of last follow-up, serum creatinine level was higher among cases (median of 182 μM [IQR, 121–231], versus 146 μM [IQR, 114–202] in controls), but the difference was not statistically different between the two groups (*p* = 0.08). The occurrence of de novo DSA was observed in cases (8/72, 11%) but not in controls (*p* = 0.01). Ten (13.8%) cases had biopsy-proven acute rejection (classified as humoral or severe mixed antibody-mediated rejection with vascular lesions), in a median delay of 191 days (IQR: 44–457). Treatment of these acute rejection episodes consisted in methylprednisolone, plasma exchanges and intravenous polyvalent immunoglobulins, with the addition of rituximab in two cases. As a comparison, in the controle group, only 3 (4.2%) patients developed acute rejection after the index date (both antibody-mediated). Overall, survival without rejection was significantly lower among cases (*p* = 0.03) (Fig. 1a).

During follow-up, 13 patients returned to dialysis (8 cases versus 5 controls, *p* = 0.07, Fig. 1b), and 4 deaths were recorded without any difference between the two groups. Event-free survival did not differ between the two groups (*p* = 0.07).

### Risk factors for NoV diarrhea

In multivariate analysis, diabetes as baseline characteristic and lymphopenia below 1000/mm^3^ at the time of diagnosis were the two risk factors statistically associated with HuNoV infection (OR 6.4 [IQR: 1.2–35], *p* = 0.03 and OR 3.8 [IQR: 1.2–12], *p* = 0.02 respectively) (Table 3). On the contrary, ciclosporin treatment was associated with a reduced risk of HuNoV diarrhea (OR = 0.34 [IQR: 0.1–0.9], *p* = 0.03). Serum gamma-glublin level below 6.5 g/L at the time of diagnosis showed values closed to statistical significance both in univariate and multivariate analysis.

**Table 3:** Risk factors for Norovirus infection

Variable	Univariate analysis		Multivariate analysis	
	Odds ratio [IQR]	*p* (Wald’s test)	Odds ratio [IQR]	*p* (Wald’s test)
Diabetes	7.5 [1.7–32.8]	0.007	6.4 [1.2–35]	0.03
Induction therapy with ATG	2.5 [0.49–12.89]	0.273		
Maintenance therapy with CT	1.8 [0.51–2.29]	0.847		
Maintenance therapy with Tac	1.29 [0.64–2.59]	0.481		
Maintenance therapy with CsA	0.5 [0.23–1.07]	0.045	0.34 [0.1–0.9]	0.03
CMV replication	1.3 [0.57–2.96]	0.533		
Serum gammaglobulin level < 6.5 g/L	4.5 [0.97–20.8]	0.054	5.1 [0.96–26.5]	0.055
Lymphocyte count < 1000/mm^3^	2.14 [0.87–5.2]	0.096	3.8 [1.2–12]	0.02

*Abbreviations*: *ATG* Anti-Thymocyte Globulin, *CsA* Ciclosporin A, *CT* Corticosteroids, *Tac* Tacrolimus

## Discussion

In this retrospective case-control study, we describe the clinical features and long-term outcome of 72 KTR with a diagnosis of HuNoV infection.

In our population, the frequency of NoV infection was 7.3%. Data regarding the overall incidence of HuNoV infection following KT are scarce. Indeed, diarrhea is a frequent symptom occurring in up to 53% of KTR [21], but most cases do not require hospitalization and/or undergo microbiological testing. Among KTR with diarrhea, recent studies evaluated the incidence of HuNoV infection between 15 to 35% [16, 22]. The frequency we observed, although consistent with preexisting data, is probably under-estimated since some patients with limited symptoms may not have been tested for HuNoV in stools.

Our results show that HuNoV infection represents a late-onset infectious complication following KT as it occurred in a median time of 46.5 months after transplantation. This result is in agreement with previous studies which report a median delay between KT and HuNoV diarrhea above 3 years [17, 18], which is longer than other infectious agents responsible for acute diarrhea such as CMV or *Clostridium difficile* [15, 21]. Indeed, the patient’s risk for infection following KT is not only linked to the intensity of the immunosuppressive regimen (which is heavier in the early post-transplant period), but also to the duration of immunosuppression. Viral infections or reactivations may therefore occur in the late post-transplantation period [4].

Clinical characteristics of HuNoV infection in our population did not differ from those described in available studies in SOTR [22]. Weight loss was observed in nearly two thirds of the cases, and was associated with severe dehydration leading to frequent acute kidney injury (60% of the patients). After diagnosis, in the absence of efficient specific therapy, immunosuppression was tapered in 93% of the patients. In agreement with previous published studies [23], we chose first to either withdraw or decrease MMF in most patients. Several studies have evaluated the administration of oral or systemic immunoglobulin with conflicting results [16, 24]. In our cohort, seven patients received intravenous polyvalent immunoglobulins due to the severity of the symptoms, and all of them had resolution of the symptoms without any further recurrence. Before the advent of possible antivirals for the treatment of HuNoV [25], systemic administration of human immunoglobulin may favor the outcome of diarrhea in the most severe patients, especially in case of underlying profound hypogammaglobulinemia.

Few studies have evaluated the long-term impact of HuNoV infection on graft function among KTR, mostly due to the short duration of follow-up. In a recent monocentric retrospective study, the incidence of renal dysfunction among KTR with diarrhea was similar between NoV and non-NoV cases at 1 month and 1 year, but was not evaluated further [15]. In our study with a median follow up of 24 and 32 months respectively in cases and controls, we observed that KTR with a diagnosis of HuNoV infection developed significantly more frequently de novo DSA than controls, and had an increased incidence of acute rejection (13.8% versus 4.2%, *P* = 0.03). However, overall graft survival did not differ between the two groups (Fig. 1), probably due to the rapid treatment of acute rejection. It is however noteworthy that patients with HuNoV infection suffer an accelerated decrease of estimated GFR (- 9 ml/min/1.73 m^2^ within 23 months versus – 4 ml/min/1.73 m^2^ in the control group). In a recent study by Rolak et al., graft survival after HuNoV diarrhea did not significantly differ compared to a control group (contrary to *Clostridioides difficile* infection), but there was a trend towards a lower graft survival [17]. This trend is confirmed by the present study, and should alert physicians to closely monitor kidney function following an episode of NoV.

In this matched case-control study, we identify the presence of diabetes and lymphopenia below 1000/mm^3^ as independent risk factors for HuNoV infection among KTR. Diabetes has already been described as a condition associated with several infection following KT [4], but our study is the first to describe its association with HuNoV diarrhea. Lymphopenia is a well-known factor associated with both viral and bacterial infections, and in a recent cohort study including 538 KTR, high blood lymphocyte count (CD4^+^ T > 500/mm^3^) at the time of transplant and during follow-up were protective factors against opportunistic infections [26]. Besides lymphopenia, patients with HuNoV infection had multiple other markers of immunosuppression. We observed that 5 (7%) of them had previous severe OI (including nocardiosis, tuberculosis or invasive aspergillosis) and, at the time of diagnosis, 14 (20.6%) had CMV plasma reactivation and 10 (13.9%) serum gamma-globulin levels below 6.5 g/L. Several studies have suggested a link between hypogammaglobulinemia and the risk of post-transplant infectious complications but in these studies, the prevalence of hypogammaglobulinemia decreased along time and concerned less than 5% of the patients beyond 6 months post-transplantation [27, 28]. In our study, hypogammaglobulinemia persisted for more than 3 years and was severe in 7 cases (less than 5 g/L), leading to the prescription of substitutive polyvalent immunoglobulins. Taken together, these data confirm that over-immunosuppression is a major risk factor for the occurrence of NoV infection.

Our study has a number of limitations. First, we conducted a single-center study and due to homogeneous immunosuppression protocols among the study population, we could not evaluate the impact of the immunosuppressive regimen on the occurrence of NoV diarrhea. Second, our statistical analysis lack sufficient power due to missing data in patients’ files and the small number of cases. Other risk factors for HuNoV diarrhea following KT may have been missed. Finally, HuNoV cases were retrospectively identified using microbiological database. The frequency of HuNoV infection in our cohort is therefore probably underestimated since some KTR with mild symptoms who did not have microbiological stool testing may have been missed. As a consequence, only the most severe cases are described and the impact of HuNoV infections on long term graft function could have been overestimated. However, in our center, KTR undergo prompt microbiological explorations in case of acute diarrhea, including norovirus testing, especially to rule out bacterial or CMV infection who could require immediate treatment. Few cases were probably omitted and our results confirm that in case of acute severe diarrhea, norovirus etiology may impact long term graft function.

In conclusion, we identified pre-transplant diabetes and lymphopenia as significant risk factors for HuNoV infection following KT, and show a higher incidence of antibody-mediated rejection and de novo DSA after the infection. These results emphasize the need for personalized immunosuppressive regimens in at-risk patients, and for reinforced preventive strategies to preserve graft function.

## Data Availability

The datasets generated and analysed during the current study are not publicly available due the local policy of the Biostatistics and Medical IT department but are available from the corresponding author on reasonable request.
